# Assessing the Measurement Invariance of the Inventory of Callous-Unemotional Traits in School Students in China and the United Kingdom

**DOI:** 10.1007/s10578-020-01018-0

**Published:** 2020-06-23

**Authors:** Jennifer L. Allen, Yiyun Shou, Meng-Cheng Wang, Elisabeth Bird

**Affiliations:** 1 Department of Psychology, University of Bath, Bath, UK; 2 Research School of Psychology, Australian National University, Canberra, Australia; 3 Department of Psychology, Guangzhou University, Guangzhou, China; 4 Department of Psychology and Human Development, University College London, London, UK

**Keywords:** Callous-unemotional traits, Psychopathy, Gender, Measurement invariance, Factor structure

## Abstract

The current study investigated the measurement invariance of the Inventory of Callous-Unemotional Traits in school-attending youth in the UK (N = 437) and China (N = 364). The original 24-item ICU and five shortened versions proposed in previous studies were tested and compared using confirmatory factor analysis in the UK sample. Results indicated that the original ICU was a poor fit in the UK sample. A shortened, 11-item version (ICU-11) featuring two factors (Callousness and Uncaring) provided the best fit and was invariant across gender in both the UK and Chinese samples. Comparisons of the ICU-11 in UK and Chinese school children revealed a similar item-factor combination and factor loadings, but different item thresholds. Findings indicate that the ICU-11 may be a preferable alternative to the original version, but that average ICU-11 scores may have a different meaning in the UK and China.

## Introduction

Callous-unemotional (CU) traits are characterized by a lack of guilt and empathy, low concern for performance and restricted or shallow emotions [1]. High levels of these traits demarcate an important subgroup of antisocial children who show more severe, varied and persistent antisocial behaviour [2, 3]. Children with CU traits show unique correlates in emotional, cognitive, and biological domains such as impaired recognition and responses to fear or distress cues [4, 5]. Evidence also suggests that these children are at risk for poorer response to treatment [6]. The presence of unique correlates, poor prognosis and reduced responsiveness to treatment in children with elevated CU traits highlights the need for assessment measures that demonstrate equivalence across children of different genders and in different cultures. There is growing evidence that CU traits is associated with significant impairment in the school setting, including disruptive behaviour, problematic relationships with teachers and peers, and poor academic performance [7–9]. Therefore, the validation of a brief measure of CU traits would facilitate research aimed at informing school-based intervention, and reduce the assessment burden on research participants.

The Inventory of Callous-Unemotional Traits (ICU) [10] is a commonly used measure of CU traits in research. It was designed for use in community samples and features youth self-report, parent and teacher versions. Earlier measures such as the Antisocial Process Screening Device (APSD) [11] assessed CU traits as one dimension of the broader construct of psychopathy and hence featured a limited number of items for the CU scale. The ICU was developed to provide a more comprehensive assessment of CU traits and to address the limitations of the APSD, including poor construct validity and internal consistency [12]. The ICU was constructed from the four items of the APSD CU traits scale that consistently loaded on the CU traits factor in clinical and community samples [13]. A 24-item scale was produced by expanding on each of these four items to encompass an additional five items matched for content. The ICU has shown good validity in children and adolescents, different types of samples and language translations [14].

### Factor Structure of the Inventory of Callous-Unemotional Traits (ICU)

The ICU was originally constructed to assess a unidimensional model of CU traits, but the initial planned study identified a bifactor model featuring one higher-order factor including all items (ICU total score) and three lower order factors: Callousness, Uncaring and Unemotional [12]. Several studies accepted this bifactor model on the basis that it showed a superior fit to either a unidimensional model or an intercorrelated three-factor model without a higher-order factor [15–17]. In general, there is limited support for the bifactor model as accurately representing the structure of the ICU, with fit statistics generally ranging from poor to unacceptable (see [18] for a review). In particular, the Unemotional scale does not appear to tap into the same construct as the other items of the ICU, shows poor internal consistency and poor criterion validity with well-established correlates of CU traits [14]. This has led researchers to argue that some or all items from the Unemotional scale should be removed from future revisions of the ICU [19, 20]. Others, however, have argued that the Unemotional scale is important when considered in combination with the two other scales in its contribution to the overarching CU construct [21].

Several research groups have attempted to refine the ICU by proposing shortened versions featuring a subset of items from the original ICU. Houghton et al. [22] developed a 16-item self-report version of the ICU (ICU-16) in 7 to 12-year-old Australian school children. A two-factor model comprising Callousness (8 items) and Uncaring factors (8 items) and featuring eight pairs of correlated errors had a marginally acceptable fit (χ^2^ = 221.63, CFI = 0.90, RMSEA = 0.07). Gao and Zhang [23] found support for a 13-item version of the self-report ICU (ICU-13). The ICU-13 had acceptable internal consistency and consisted of two factors: Callousness (7 items) and Uncaring (6 items). Hawes et al. [19] developed a 12-item form of the parent report ICU (ICU-12) including Callousness (7 items) and Uncaring (5 items) using item response theory (IRT). The ICU-12 showed adequate short-term retest reliability, high internal consistency and good discrimination along the continuum of the CU construct. The self-report version of the ICU-12 has also shown good construct validity in youth recruited through schools and juvenile detention centres [24–26].

Ray et al. [27] found support for a unidimensional, 10-item self-report measure of CU traits (ICU-10) developed from IRT analyses in a large sample of antisocial adolescents. The ICU-10 included 7 items from the Callousness factor and three from the Uncaring factor. The ICU-10 showed good internal consistency (alpha = 0.78) and 6-month retest reliability (*r* = 0.59). The findings of Ray et al. also suggested that the two-factor structure found in many studies may reflect a method factor and/or differences in item severity, as all of the ICU items on the Uncaring factor are negatively worded, and only one item on the Callousness factor is positively worded. Higher ratings on positively worded items (higher CU traits) were more likely to be rated in the lower response categories, and positively worded items discriminated best at higher levels of CU traits. Pecchoro et al. [25] recently compared the unidimensional ICI-10 and the two-factor ICU-12 in Portuguese male detained adolescents and found support for both shortened versions in terms of their factor structure and internal consistency. However, the ICU-12 had a much better fit (χ^2^ = 2.15, CFI = 0.97, RMSEA = 0.07) than the ICU-10 (χ^2^ = 3.33, CFI = 0.95, RMSEA = 0.10).

Finally, Colins et al. [28] found that an 11-item model (ICU-11) which excluded the only item retained from the unemotional factor in the ICU-12 (‘does not show emotions’) achieved a better fit (χ^2^ = 58.51, CFI = 0.96, RMSEA = 0.06) compared to the original 24-item structure (χ^2^ = 723.08, CFI = 0.69, RMSEA = 0.11) in 191 female detained adolescents. The two-factor ICU-11 also showed acceptable to good internal consistency for the total and subfactor scores (alphas 0.72 to 0.76) and improved criterion validity than the original ICU. Overall, shortened measures of the ICU show great promise as efficient, reliable and valid measures of CU traits. Research on the full version of the self-report ICU in different European countries (e.g., Belgium, Germany) and the United States suggests that there may be mean differences in the severity of CU traits across Western countries [12, 17, 29, 30]. Given differences in the severity, strength of item ratings and the stability of symptoms across different dimensions of psychopathic traits in adults in North America and the UK [31], it is important to examine the measurement invariance of the original and shortened versions of the self-report ICU for children in the UK.

### Gender Invariance of the Inventory of Callous-Unemotional Traits

It is important to examine the invariance of the ICU across gender given evidence suggesting that the factor structure of CU traits may differ for boys and girls [32]. Moreover, boys show more severe CU traits and comorbid externalizing problems, while girls have more severe internalizing problems [14]. Boys with elevated CU traits also show greater impairment than girls in their social and academic functioning [8, 9, 33]. Gender differences in CU traits may be due to biological differences or socialization processes [25]. For example, socialization processes that influence empathy, temperament (e.g., behavioural inhibition, inhibitory control) and emotional expression may differ for boys and girls [34, 35]. To date, research has uniformly found support for the invariance of the ICU across gender [12, 22, 23, 36], but this remains to be tested in a UK sample.

### Measurement Invariance of the Inventory of Callous-Unemotional Traits in China

Several recent studies have compared the original and five shortened forms of the ICU (i.e., ICU-10, ICU-11, ICU-12, ICU-13 and ICU-16) in Chinese populations. Wang et al. [37] found that the two-factor ICU-11 featuring Callousness and Uncaring dimensions was the best-fitting model, showing an excellent overall fit in children attending a mainstream primary school. The ICU-11 was invariant across informant (self-report, parent-report, teacher-report) and occasions, with marginal internal consistency for the subfactor scores. Wang et al. [18] also found that the self-report ICU-11 provided the best fitting model in Chinese university undergraduates. Similarly, Zhang et al. [38] found that the two-factor, self-report version of the ICU-11 formed the best fitting model of the original and five shortened versions in Chinese detained adolescents.

Psychopathy/CU traits has predominantly been conceptualized through European and American views on personality, self-concept and social norms [39]. CU traits may carry a higher degree of severity and impairment within collectivist, East Asian cultures where a stronger emphasis is placed on interpersonal connectedness and prioritizing the needs of the group/others above one’s own self-interest compared to more individualistic Western nations [40]. Items assessing CU traits may also be interpreted differently by East Asians due to cultural ‘display rules’ around emotion. For example, Fung et al. [41] found that parents in Hong Kong rated children higher on the APSD CU traits scale compared to US norms. The authors reasoned that a Chinese cultural norm around the suppression of emotion resulted in inflated levels of ‘unemotional’ traits. An East–West comparison of the ICU is currently lacking, despite its status as the most commonly used measure in CU traits research. Given recent evidence for the utility of the ICU in Chinese youth [37, 38], a cross-cultural comparison between school children from a Western nation (UK) and China is timely.

### The Present Study

The first aim of the present study was to test the factor structure of the original and five shortened versions of the ICU in a UK sample. We focused on the UK sample given that previous research has examined this issue in Chinese primary school children, finding that the ICU-11 was the best fitting model [37]. The second aim was to (i) test the invariance of the best-fitting model across gender in the UK and Chinese samples, and (ii) examine its measurement invariance between the UK and Chinese samples. Finally, we examined the internal consistency of the best-fitting model of the ICU in both nations. In the present study, culture is defined as a set of attitudes, beliefs and behaviours common to a group of people [42, 43], as opposed to race. Accordingly, both samples included minority ethnicity children in order to capture a true reflection of the UK and Chinese school contexts. Based on prior research [19, 28, 37] we expected the two-factor ICU-11 and/or ICU-12 to provide the best-fitting models. We also predicted that the best-fitting model would be invariant across gender and show good internal consistency in the UK and Chinese samples. Based on previous research [37] showing that ICU-11/12 also fit well in Chinese children (the fit indices of the two models are both excellent), we should expect at least configural invariance across two samples.

## Method

### Participants

UK participants included 437 children attending Years 7 to 9 of a state secondary school in the east of England. Children were aged 11 to 14 years (50.3% girls; *M* = 12.50 years, SD = 0.96). Most children were White (95%), with the remainder identifying as Asian (*n* = 1), Mixed White and Asian (*n* = 6), Black (*n* = 6) or Mixed Black and White (*n* = 4). Nearly a quarter of the sample (23%) had English as an additional language, 16% belonged to single-parent families and 11% were in receipt of free school meals. All children were fluent in English as a requirement for participation. See Bird et al. [8] for more detailed information about the UK sample. The Chinese sample included 364 children in Grades 4 to 6 of a mainstream primary school in Guangdong in China. Children were aged 10 to 13 years (49% girls; *M* = 10.77, SD = 0.77) and almost all were of Han ethnicity (99%). Most parents had attained an educational qualification following high school (71.5%) and only 3% of students belonged to single-parent families. The Chinese sample comes from an ongoing longitudinal study and this wave has not been previously published. Data from different waves in this sample have been tested, with further detail about sample characteristics contained in Wang et al. [37, 44].

### Measures

#### *Inventory of Callous-Unemotional Traits* (ICU) [10]

Child report of callous-unemotional (CU) traits was assessed using the 24-item ICU. Items are rated on 4-point scale from 0 ‘not at all true’ to 3 ‘definitely true’. The reliability and validity of the youth self-report version of the ICU has been supported across multiple translations, including Mandarin Chinese [12, 30, 37]. Different items were selected for analysis, depending on the model tested (see Table 3 for model specifications). Translations of the ICU can be obtained from the website of the measure developer, Paul Frick: https://labs.uno.edu/developmental-psychopathology/ICU.html.

#### Sociodemographic Characteristics

Children in both samples reported their age, gender, ethnicity and membership of a two-parent or single parent family. English as an additional language and receipt of free school meals was assessed in the UK sample. Free school meal eligibility is used as a proxy for socioeconomic disadvantage in England [45].

### Procedure

#### UK Sample

Study procedures were approved by the university ethics board prior to data collection. School approval, along with parent opt-out and child written informed consent was obtained. Students independently completed a brief questionnaire to obtain basic demographic information and the self-report version of the ICU in their classroom during regular school hours, as part of a larger questionnaire battery. Students were instructed that they could return the questionnaires incomplete if they did not wish to take part; and were not offered any incentives for participation. Students had the opportunity to ask the researcher any questions during questionnaire completion, including clarification of the wording of questionnaire items. Two participants were missing values for the ICU and were thus excluded from the analyses.

#### Chinese sample

Following the receipt of university ethics board approval, the approval of the head of school and informed written parental consent was obtained. Verbal assent of children was obtained prior to study commencement. Only children who agreed to participate were asked to complete the self-report questionnaires. Children completed the self-report version of the ICU as well as other questionnaires (not featured in this present study) during a class session during normal class time. Children who completed the questionnaires were each paid by gift vouchers worth approximately 15 Yuan or US$2 for their participation.

### Data Analysis

#### Step 1: Confirmatory Factor Analyses (CFA)

We first used a series of CFAs to examine the 3-factor model of the original ICU (24-item) and 1 or 2-factor models of the five shortened versions used the “lavaan” package [46] in R program (version 3.6.2). As the ICU items only had 4 response categories, we used the robust weighted least-squares with mean and variance adjustment (WLSMV) estimator to minimise estimation bias. The main fit indices used to compare different models included: root-mean-square error of approximation (RMSEA; ≤ 0.08 indicates an acceptable model fit), Tucker-Lewis index (TLI; ≥ . 90 indicates an acceptable model fit), comparative fit index (CFI; ≥ . 90 indicates an acceptable model fit), and Bayesian Information Criterion (smaller values indicate a better and more parsimonious model) [47]. A model is considered as superior to the other models if all, or the majority of its fit indices were better than those of the other models.

#### Step 2. Measurement Invariance (MI)

The factor model that had the best model fit was used to examine the measurement invariance (MI) of the ICU across gender in the UK and Chinese samples separately, and across the UK and Chinese samples. Three levels of MI were tested using multiple group CFA. The first was configural invariance, where the model does not have constraints placed on any parameters. Good model fit indicates that the item-factor structure is similar across groups. The second was metric invariance, where a weakly constrained model with item factor loadings set to be equal across groups was estimated. Metric invariance is supported if the model fit of the weakly constrained model is similar to the first freely estimated model. The third level was scalar or strong factorial invariance, where a strongly constrained model with the item thresholds further equally constrained was estimated. Strong invariance is supported if the model fit of the strongly constrained model is similar to the second weakly constrained model.

The comparison of the models was conducted using multiple indicators: chi-square difference test, change in indices such as CFI (ΔCFI), TLI (ΔTLI) and RMSEA (ΔRMSEA). Invariance hypothesis is supported by nonsignificant chi-square difference test, a ΔCFI and a ΔTLI smaller than 0.01, and ΔRMSEA smaller than 0.005 [48].

#### Step 3. Reliability Analyses

Both Cronbach’s alphas and McDonald’s hierarchical omega based on polychoric correlations were used to assess the reliability of the ICU scores. Reliability indices were calculated for both the total score and the subscale scores for the original and the best short version of the ICU. Mean inter-item correlations (MICs) were also reported given the reliance of α on the number of items. MICs are independent of scale lengths and are considered acceptable if they fall in the range of 0.15 to 0.50 [47].

## Results

### Confirmatory Factor Analyses

Multiple fit indices for the original 24-item ICU and five shortened versions are presented in Table 1. The fit for the original ICU model was not satisfactory for most indices, while the bi-factor model only achieved the acceptable value for the CFI. Of the shortened versions, only the ICU-11 and ICU-12 showed an acceptable fit for all fit indices (e.g., CFIs > 0.90 and TLIs > 0.90) and both were a much better fit to the data provided by the UK sample than the other models. The ICU-11 had better CFI and TLI values, as well as BIC, and slightly lower RMSEA than the ICU-12. Thus, the ICU-11 was determined to be the best fitting model. The factor loadings for the original ICU and the shortened versions are displayed in Table 2. The factor loadings for the ICU-11 all fell above the generally recommended threshold of 0.40 [49], ranging from 0.43 to 0.81. Considering model fit across multiple indices and factor loadings, we selected the ICU-11 as the best model to examine gender invariance in the UK and Chinese samples separately, and measurement invariance across the nations of the UK and China.

**Table 1:** Goodness-of-fit indices for the tested models in the confirmatory factor analysis

Model	χ2	*df*	CFI	TLI	RMSEA [90% CI]	BIC
ICU-24	844.55	249	0.86	0.84	0.07 [0.07, 0.08]	24,521.17
ICU-24 Bifactor	617.36	228	0.91	0.89	0.06 [0.06, 0.07]	24,402.67
ICU-10	335.71	35	0.89	0.86	0.14 [0.13, 0.15]	10,124.24
ICU-11	98.61	43	0.96	0.95	0.06 [0.04, 0.07]	10,991.59
ICU-12	114.86	53	0.95	0.94	0.05 [0.04, 0.07]	12,051.25
ICU-13	282.81	64	0.93	0.92	0.09 [0.08, 0.10]	12,680.56
ICU-14	513.28	89	0.88	0.86	0.11 [0.10, 0.11]	14,780.55
ICU-16	532.68	103	0.88	0.86	0.10 [0.09, 0.11]	15,824.39

*df* degrees of freedom, *CFI* comparative fit index, *TLI* Tucker-Lewis index, *RMSEA* root mean square error of approximation, *CI* confidence interval. The best-fitting model is in bold

BICs were calculated by using maximum likelihood estimators with adjusted means

**Table 2:** Standardized factor loadings of ICU-11 in the UK sample

Item	Factor loadings
Callousness	
4. I do not care who I hurt to get what I want	0.72
9. I do not care if I get into trouble	0.64
11. I do not care about doing things well	0.64
12. I seem very cold and uncaring to others	0.56
18. I do not feel remorseful when I do something wrong	0.43
21. The feelings of others are unimportant to me	0.49
Uncaring	
5. I feel bad or guilty when I do something wrong (R)	0.52
8. I am concerned about the feelings of others (R)	0.64
16. I apologize (say ‘I am sorry’) to persons I hurt (R)	0.80
17. I try not to hurt others’ feelings (R)	0.71
24. I do things to make others feel good (R)	0.47

*ICU* Inventory of Callous-Unemotional Traits. (R) indicates a reverse-scored item

All factor loadings were significant at the .001 level

### Internal Consistency of ICU Scores

Alphas and omegas for the original ICU scale and the ICU-11 for both the UK and Chinese samples are presented in Table 3. The MICs were acceptable for both ICU-24 and ICU-11 total and subscale scores for both the UK and Chinese samples. The reliability indices—alphas and omegas—were acceptable for both ICU versions except for the unemotional scale of the ICU-24.

**Table 3:** Internal consistency of the ICU-24 and ICU-11 in the United Kingdom and Chinese samples

	UK			Chinese		
	Alpha	Omega	MIC	Alpha	Omega	MIC
ICU-24	0.83	0.87	0.17	0.87	0.83	0.19
Unemotional	0.66	0.59	0.28	0.52	0.42	0.18
Callousness	0.78	0.74	0.25	0.77	0.73	0.23
Uncaring	0.82	0.83	0.37	0.83	0.78	0.38
ICU-11	0.80	0.81	0.27	0.77	0.82	0.23
Callousness	0.75	0.75	0.33	0.70	0.76	0.28
Uncaring	0.76	0.71	0.39	0.65	0.68	0.27

*ICU* inventory of callous-unemotional traits

### Measurement Invariance Across Girls and Boys in the UK and Chinese Samples

Given that the ICU-11 was the best fitting model, we then proceeded to examine the measurement invariance of this shortened version across girls and boys in the UK and Chinese samples. Model fit indices for the measurement invariance of the ICU-11 for girls and boys in the UK and China are presented in Table 4. In the UK sample, examination of configural invariance indicated that the ICU-11 fit both groups well. The metric invariance model was then tested by constraining the strength of factor loadings equally across both genders. The results indicated that there were no significant differences in the strength of factor loadings for boys and girls (ΔCFI less than 0.01). Similarly, the test of scalar invariance showed that item thresholds were similar across groups. Strict invariance was also supported in terms of both ΔCFI and ΔTLI. Since strict invariance is satisfied, we examined the gender differences in the means of the latent traits. Results demonstrated that there was no significant difference in the latent mean of the callousness trait factor (mean difference =  − 0.226, SE = 0.126, p = 0.073). However, girls had a significant lower mean for uncaring traits than boys (mean difference =  − 0.152, SE = 0.066, *p* = 0.020). For the Chinese sample, metric and scalar invariances between boys and girls were generally met.

**Table 4:** Measurement invariance of the ICU-11 across gender

	S-Bχ^2^	*df*	CFI	TLI	RMSEA [90% CI]	Δχ^2^	Δ*df*	*p*	ΔCFI	ΔTLI
United Kingdom sample										
Configural invariance	157.24	86	0.95	0.94	.06 [.05, .08]					
Metric invariance	169.98	95	0.95	0.94	.06 [.05, .08]	12.74	9	.175	.000^a^	.000^a^
Scalar invariance	183.55	115	0.95	0.95	.05 [.04, .07]	13.57	20	.852	.000^a^	.000^a^
Chinese Sample										
Configural invariance	172.67	86	0.951	0.937	.075 [.058, .091]					
Metric invariance	198.51	95	0.941	0.932	.078 [.062, .093]	18.75	9	.027	.01	.004
Scalar invariance	187.84	115	0.958	0.960	.059 [.043, .074]	-	-	-	.000^a^	.000^a^

*df* degrees of freedom, *S-Bχ*^*2*^ Satorra-Bentler chi-square, *CFI* comparative fit index, *TLI* Tucker-Lewis index, *RMSEA* root mean square error of approximation, *CI* confidence interval

^**^p < .001

^a^The increasing CFI and TLI with a more constrained model are caused by the result that the Δ*df* is greater than Δχ^2^

### Measurement Invariance across Chinese and UK School Children

Before carrying out the measurement invariance tests, we first examined whether the ICU-11 would be the best fitting model among the different models for the current Chinese sample. Table 5 displays the model fit results and it is clear that ICU-11 had the best model fit among all models in terms of all fit indices.

**Table 5:** Goodness-of-fit indices for the tested models in the confirmatory factor analysis—Chinese sample

Model	χ2	*df*	CFI	TLI	RMSEA [90% CI]	BIC
ICU-24	677.97	249	0.89	0.87	0.07 [0.06, 0.08]	18,024.19
ICU-24 Bifactor	617.36	228	0.91	0.89	0.06 [0.06, 0.07]	24,402.67
ICU-10	151.55	35	0.93	0.91	0.10 [0.08, 0.11]	7328.99
ICU-11	112.93	43	0.96	0.95	0.07 [0.05, 0.08]	7869.86
ICU-12	137.65	53	0.95	0.94	0.07 [0.05, 0.08]	8797.73
ICU-13	244.18	64	0.92	0.91	0.09 [0.08, 0.10]	9647.94
ICU-14	295.61	89	0.92	0.91	0.08 [0.07, 0.09]	10,771.15
ICU-16	321.35	103	0.93	0.92	0.08 [0.07, 0.09]	11,374.31

The best-fitting model is in bold. BICs were calculated by using maximum likelihood estimators with adjusted means

*df* degrees of freedom, *CFI* comparative fit index, *TLI* Tucker-Lewis index, *RMSEA* root mean square error of approximation, *CI* confidence interval

The ICU-11 was then used to test the measurement invariance in UK and Chinese samples (see Table 6). The configural invariance model provided a good fit to the data in terms of all fit indices (CFI = 0.96, TLI = 0.95, RMSEA = 0.06). The good model fit for the configural invariance model indicates the item-factor combination is similar between the two groups. There was a significant difference in the model fit between the metric invariance model and the configural invariance model. Inspecting the modification indices suggests that item 21 “The feelings of others are unimportant to me” had a stronger loading on the Callousness factor for the Chinese sample than for the English sample. Allowing this item to have a freely estimated loading across groups substantially improved model fit and resulted in little difference in the model fit between the metric invariance model and the configural invariance model (ΔCFI = 0.006).

**Table 6:** Measurement invariance of the ICU-11 across the Chinese and United Kingdom samples

	S-Bχ^2^	*df*	CFI	TLI	RMSEA [90% CI]	Δχ^2^	Δ*df*	*p*	ΔCFI	ΔTLI
Configural invariance	211.06	86	0.96	0.95	0.06 [0.05, 0.07]					
Metric invariance	256.49	95	0.95	0.94	0.07 [0.06, 0.08]	45.43	9	< .001	.012	.009
Partial metric^a^	236.77	94	0.95	0.95	0.06 [0.05, 0.07]	25.71	8	.001	.006	.002
Scalar invariance	394.19	114	0.91	0.91	0.08 [0.07, 0.09]	157.42	20	< .001	.047	.036
Partial scalar invariance^b^	263.54	103	0.94	0.93	0.07 [0.06, 0.08]	26.77	9	.002	.016	.013

*df* degrees of freedom, S-Bχ^2^ Satorra-Bentler chi-square, *CFI* comparative fit index, *TLI* Tucker-Lewis index, *RMSEA* root mean square error of approximation, *CI* confidence interval

**p < .001

^a^One item was allowed to have a free loading parameter (Item 21)

^b^Four items were allowed to have free threshold parameters (Items 5, 11, 16 and 18)

In contrast, the model fit dropped substantially at the scalar level (CFI difference = 0.047), suggesting the presence of significant differences in thresholds between the two groups. Inspecting the modification indices suggests that there were several items showing significant differences in item thresholds between the two groups. This prevents further investigation using other invariance tests (e.g., strict and latent mean invariance). The model fit was only improved after four items (more than one third of the total number of items) were allowed to have free parameters (i.e., item thresholds can differ across the two groups) (CFI = 0.975). These results indicate that the mean scores of the ICU are not directly comparable between the Chinese and the UK samples. However, cross-cultural comparisons on how ICU scores are correlated with external criteria are feasible (e.g., investigating if regression coefficients are moderated by culture when using ICU scores).

## Discussion

The first aim of this study was to examine the factor structure of the original and five shortened versions of the ICU in a UK sample. The second aim was to investigate the measurement invariance of the best-fitting model in the UK sample, and then use this model to examine its measurement invariance (i) across gender, and (ii) between the UK and Chinese samples of school children. Our results indicated that the two-factor ICU-11 featuring Callousness and Uncaring dimensions produced the best fit and was invariant across girls and boys in the UK sample. The ICU-11 had a similar item-factor combination and factor loadings for the UK and Chinese samples; however, item thresholds were not equivalent across groups, indicating that it is not meaningful to compare average scores for school students in these two nations.

### Confirmatory Factor Analysis

The current study tested and compared the original 24-item ICU and five different shortened versions. Consistent with past studies (see [18] for a review), the three-factor model of the original ICU was a poor fit in the UK sample. The strongest support was found for the two-factor second-order model, consistent with past studies examining the item-factor structure of the ICU [28, 37, 38]. The ICU-11 was the best fitting model, outperforming the ICU-12 on two indices (CFI and TLI values), but with slightly lower RMSEA than the ICU-12. Both the ICU-11 and ICU-12, however, were a much better fit for the data than the other shortened versions. Past research has also shown the strongest support for the two-factor, 11- and 12-item versions using different sample types [19, 24, 25, 28, 37]. The ICU-11 and the ICU-12 contain a two-factor structure and similar items—the only difference between the two versions is that the ICU-12 includes item 6 ‘do not show emotions’—therefore it is not surprising that they yielded similar fit results.

The ICU-11 does not include any items from the Unemotional scale, suggesting that these items index a construct that is distinct from the Callousness and Uncaring dimensions. Furthermore, the internal consistency of the ICU-24 and ICU-11 in the UK and Chinese samples was acceptable or good for all total and subfactor scores, except for the unemotional factor of the ICU-24. The poor construct validity, low internal consistency and poor external validity of the Unemotional scale has been replicated in many studies [14]. The Unemotional scale items may not be precisely measuring emotion as related to CU features. Rather than a global reduction in affect, the intensity of emotion for children with CU traits appears to differ across emotion types. CU traits are associated with reduced guilt, fear and sensitivity to others’ distress, while anger appears to be experienced more intensely [6]. It is challenging to assess the affective features of CU traits given the complexity of emotions that are rapidly elicited, experienced and expressed in constantly changing sequences of social interaction in the few short words permitted within a questionnaire format [50]. Self-report questionnaire ratings have shown weak associations between CU traits and positive affect [51], but studies examining positive emotion in context found that children with elevated CU traits displayed intense positive affect (e.g., joy, excitement) when engaging in risk-taking activities, bullying others, or witnessing others’ conflict [52], and reported feelings of pride when failing to reciprocate to others who have helped them [53]. The affective features of CU traits may necessitate multiple assessment methods, including ‘other’-informant interviews and experimental tasks.

Similar to past research [36], latent means were lower for girls on uncaring traits, but there was no gender difference for the latent means for callousness. The factor structure and strengths of factor loadings of the ICU 11 were equivalent across boys and girls, consistent with past research examining the gender invariance of the ICU [12, 22, 23, 36]. Furthermore, results indicated that mean scores of the ICU-11 might be directly comparable for both genders. Therefore, while there is strong evidence for gender differences in terms of the severity of CU traits, degree of psychosocial impairment and patterns of comorbidity [12, 52–54], studies examining the gender invariance of the ICU have uniformly found that ICU scores are equivalent across boys and girls. Given that boys show greater social and academic impairment than girls in the school setting [8, 33, 36], it is important that measures demonstrate equivalence across gender to ensure the accurate identification of at-risk children for school-based intervention.

Examination of the measurement invariance of the ICU-11 across nations indicated that it has a similar item-factor structure and factor loadings for Chinese and UK school students. Only one item “The feelings of others are unimportant to me” showed a stronger association with the Callousness factor in the Chinese sample than the UK sample. This may reflects a cultural difference in that Chinese culture more promotes Zhongyong thinking style (encourages individuals to consider others’ thinking and a willingness to ‘step back’ during conflict to promote interpersonal harmony) and vertical collectivism (willingness to sacrifice one’s own benefits for the sake of the group benefits) [39].

A strict invariance model of the ICU-11 only achieved adequate model fit when item intercepts were freed for four items—more than a third of the total scale items. It is difficult to establish strict measurement invariance between Chinese and UK samples. This indicates that mean ICU-11 scores may not be directly comparable across UK and Chinese school children, although comparison of ICU-11 scores with reference to external criteria (e.g., antisocial behaviour, empathy) are feasible. The variation of item intercepts or thresholds may be due to UK or Chinese school children systematically rating some items much higher or lower than the other group, potentially due to a social norm. The item that displayed the largest difference was the item “I do not care about doing things well”. Chinese children were more likely to endorse option 1 (slightly true for me) while the majority of UK children endorsed the option 0 (not true at all). One possible explanation is that Chinese schools and parents usually hold high expectations for children in relation to achievement [55]. Chinese children endorsed option 1, suggesting they may perceive that their level of effort may not meet the expectations of parents and teachers. Other items that showed differences included the item “I apologize (“say I am sorry”)” to persons I hurt”, where Chinese children were more likely to score 1 or 2 (somewhat true or very true for me), while UK children were slightly more likely to score 3 (definitely true); and the item “I do not feel remorseful when I have done something wrong” where Chinese students were more likely to score 0 or 1 (not at all true or somewhat true), while UK students were slightly more 2 and 3 (very true or definitely true). This is interesting as it suggests that while UK students understand the social value in apologizing, they do not actually feel sorry. Apologizing is heavily socialized in England [56], but it may also be that the slightly older age of students in the UK sample enabled them to better understand the importance of an apology for self-presentation and the preservation of social relationships following a transgression [57], even if the apology is not ‘felt’.

Thus, while items appear to be interpreted in the same manner by UK and Chinese school children, there are cultural differences in the strength of item endorsement. Past research on CU traits in East Asian cultures have indicated differences in the severity of CU traits across nations [41]. Furthermore, CU traits assessed via the APSD failed to demonstrate significant relationships with aggression and antisocial behaviour in East Asian children [58, 59], despite the status of these constructs as well-established correlates of CU traits in Western samples [1]. East–West comparisons of adult psychopathic traits has provided support for the universality of this construct [39]; however, research in children appears to suggest cultural variation in the manifestation of CU traits and externalizing problems. Now that the reliability and validity of the ICU-11 has been established in community and detained samples [37, 38], future work should extend investigation to understanding East–West cultural variation in the psychological processes underlying CU traits.

### Limitations and Future Directions

This study has several limitations that should be acknowledged. The UK and Chinese samples were non-referred children from a single school in each nation. It is important to validate brief measures in nonclinical samples before CU traits and antisocial behaviour becomes severe and impairing. However, our findings may not generalize to clinical, forensic or adjudicated samples due to the restricted range of CU traits likely to be present in mainstream school samples. The UK sample also featured secondary school students whose average age was slightly older than that of the Chinese primary school students. While norms for the ICU [60] indicate higher total scores for adolescents (15–17 years) compared to children (11 to 14 years), past research in children aged 11 to 14 years found that ICU scores did not differ across grade level [36]. Thus, increasing CU traits severity with age appears to be present across a larger age range that is present in the current study. Increasing CU trait severity with age has been attributed to various factors, including the effects of puberty [61], lesser ability of children to engage in self-reflection regarding their own attributes, and increased uncaring and antisocial attitudes in adolescence [22]. However, differences in the mean value of ICU does not mean that the factor structure differs across age groups. Indeed, past research indicates that while younger children had lower scores on the Uncaring scale than older children, ICU scores were invariant across child age, showing similar factor variances and factor loadings [22]. This study also focused solely on the self-report version of the ICU. Inclusion of the parent and teacher versions of the ICU would enable the examination of cross-informant invariance. Nevertheless, self-report information is crucial for gaining insight into subjective experiences that teachers and parents may be unaware of, particularly antisocial tendencies and attitudes [13, 23]. It should be noted that there are differing norms depending on the country in which the sample is tested. Future research should develop norms for the ICU-11 in different nations.

Current study findings highlight the importance of investigating the equivalence of measures across cultures, due to potential differences in item interpretation and ratings based on cultural values and perceptions of deviance from social norms. Future research should include external correlates of CU traits to better identify the construct invariance of this measure between UK and Chinese school children. Ideally, this would go beyond antisocial behaviour to encompass the emotional, cognitive and biological correlates of CU traits (e.g., reduced amygdala activation). Nevertheless, to the best of our knowledge, this study it is the first to compare the ICU in a Western and an East Asian nation. This is also the first study to examine the factor structure of the original and short forms and gender invariance of the ICU in a UK sample. The validation of a brief measure enhances our understanding of CU traits, enables greater precision in its measurement and reduces the assessment burden for future research participants.

In conclusion, this study found that a short form of the self-report ICU featuring 11 items and a two-factor structure (Callousness and Uncaring dimensions) demonstrated better construct validity than the original form. Our findings provide further support for the gender invariance of the ICU [12, 22, 23, 36] and extends prior work by showing that the 11-item, self-report version shows acceptable to good internal consistency and is invariant for boys and girls in the UK. The ICU-11 may therefore be preferred to the original form in UK children aged 11 to 14 years. There is increasing interest in CU traits and school-related risk factors [9, 33, 52]. As such, a reliable, valid and time-efficient measure of CU will facilitate research in children attending mainstream schools. The cross-cultural comparison indicated that the item-factor structure and factor loadings were equivalent for Chinese and UK school children; however, mean ICU-11 scores are not directly comparable for these two groups. The extension and replication of the current findings into other Western and East Asian nations would increase our understanding of the cultural implications of the manifestation and development of CU traits in children.

## Summary

Callous-unemotional (CU) traits are characterized by low empathy, guilt, emotionality and a lack of concern for performance. High levels of these traits are related to more varied and severe antisocial behaviour and impairment, including in the school context. There are differences in the presentation and correlates of CU traits as a function of child gender and in Western compared to East Asian cultures. This study therefore investigated the measurement invariance of the Inventory of Callous-Unemotional traits (ICU) in male and female school students in the United Kingdom (UK) and China. The original 24-item ICU, ICU-bifactor model and five shortened versions proposed in previous studies were tested and compared using confirmatory factor analysis in the UK sample. The original 24-item ICU was a poor fit for the data in both nations, while the bi-factor model only achieved the acceptable value for one index of model fit. A shortened, 11-item version (ICU-11) featuring two factors (Callousness and Uncaring) provided the best fit and was invariant across gender in both the UK and Chinese samples. The reliability indices were acceptable for both the ICU-24 and the ICU-11 except for the unemotional scale of the ICU-24. Comparisons of the ICU-11 in UK and Chinese school children revealed a similar item-factor combination and factor loadings, but different item thresholds. Findings indicate that the ICU-11 may be a preferable alternative to the original version, but that average ICU-11 scores may have a different meaning in the UK and China.
